# Case Report: Acute Kidney Failure due to Massive Envenomation of a Two-Year-Old Child Caused by Killer Bee Stings

**DOI:** 10.4269/ajtmh.20-1276

**Published:** 2021-05-10

**Authors:** Swann Geoffroy, Alexis Fremery, Yann Lambert, Christian Marty, Narcisse Elenga

**Affiliations:** 1 Service de Médecine et Chirurgie Pédiatrique, Cayenne Hospital, Cayenne, French Guiana;; 2 Service des urgences, Cayenne Hospital, Cayenne, French Guiana;; 3 Centre d’Investigation Clinique Antilles-Guyane (Inserm 1424), Cayenne Hospital, Cayenne, French Guiana;; 4 Croix-Rouge Française, Cayenne, French Guiana

## Abstract

A hybrid species of Brazilian bee has proliferated on the South American continent since 1956. We describe a “killer bee” swarm attack on a 2-year-old girl in French Guiana. The patient weighed 10 kg, and approximately hundreds of bees’ stingers were removed, that is, 10 stings/kg. Our patient survived without long-term sequelae. The management of her condition required admission into intensive care for renal failure due to acute tubular necrosis and severe rhabdomyolysis. We emphasize the importance of early medical intervention, clinical surveillance, and biological monitoring at the hospital to prevent a toxic chain reaction that could prove fatal within 72 hours.

## INTRODUCTION

“Killer bees” procured their nickname because of their exceptionally strong defensive behavior and their ability to carry out coordinated attacks involving several hundred individuals.^1^ This behavioral phenotype is transmitted through the hybridization of a relatively docile European honeybee introduced in Latin America in the seventeenth century and a more aggressive African species (*Apis mellifera scutellata*) introduced in Brazil in 1956.^2^

The latter quickly became uncontrollable, invading the American continent^3,4^ by killing or hybridizing with local bees. Thus, in the 1980s, several DNA studies showed that wild honeybees in most Latin American countries were Africanized to a high degree.^1^

Currently, this species of bee is known to cause public health problems^4–8^ due to the potential risk of systemic toxic reaction from as little as 50 stings for an adult, that is, 1 sting/kg.^9^

In French Guiana, a French overseas region partly located in the Amazon and bordering Suriname and Brazil, Hommel and Le Borgne have described several massive envenomations,^10,11^ but, to our knowledge, no attack on a child had been reported thus far on this territory.

Here, we describe the clinical symptoms and sequelae following a swarm attack by Africanized honeybees on a 2-year-old girl. The aim of this article was to explain the pathophysiological mechanisms identified and to recall the particularities for managing such a severe envenomation.

## CASE

In a village along the Maroni River in French Guiana, a 2-year-old girl was attacked by a colony of bees around 3:00 pm outside, near her home. Within 2 hours, she was admitted to a remote healthcare center with diffuse pain, diarrhea, vomiting, and cutaneous inflammation all over the body, including the face, with complete occlusion of the eyelids. Clinical examination did not reveal any hemodynamic, respiratory, or neurological deficits.

The patient weighed 10 kg, and approximately 100 bees’ stingers were removed, that is, 10 stings/kg, most of which were located on her head including the eyelids and mouth, neck, upper body, and upper limbs.

She was immediately administered 20 mg of prednisolone, 5 mg of polaramine, 150 mg of paracetamol, and a polyionic solution at 150 mL/kg/24 hours by intravenous infusion. Eighteen hours after the envenomation, she remained anuric. The first-aid doctor did not have the resources to perform a blood test on the spot and requested medical evacuation by air ambulance to a pediatric intensive care unit (ICU).

On arrival at the ICU, her blood pressure was at 100/60 mmHg, heart rate at 110 beats/minute, temperature at 37°C, oxygen saturation at 100%, and her state of consciousness was not altered. She seemed to be in pain and remained anuric.

She continued to receive 20 mg/day of prednisolone, 5 mg/day of dexchlorpheniramine, 600 mg/day of paracetamol, and hydration with a polyionic solution for the first 7 days. We also introduced 10 mg/day of furosemide for 3 days.

Her gastrointestinal symptoms ceased 36 hours after envenomation, her diuresis normalized to 5 mL/kg/hour within 72 hours, and her facial edema persisted for the first week.

The biochemical workup (Table 1) showed acute functional renal failure worsening up to day four alongside hyperkalemia, which peaked at hour 30. At the same time, increases in creatine kinase (CK) and transaminases (aspartate transferase and alanine transaminase) indicated severe rhabdomyolysis.

**Table 1:** Summary table of blood biochemistry results

Time after stings	19 hours	30 hours	3 days	4 days	5 days	7 days	9 days	12 days	16 days
Na135-145 mmol/L	129	130	130	133	133	142	140	141	137
K 3.5–5 mmol/L	7.4	8.1	5.6	4.8	3.9	4.6	4.6	4.8	4.8
Corrected K (pH = 7.16)	–	6.7	–	–	–	–	–	–	–
Urea 1.8–6.0 mmol/L	15.8	18.7	26.8	28.9	23.9	14.5	2.2	3.1	3.1
Creatinine 30–70 µmol/L	135	169	255	257	125	74	33	34	34
Creatine kinase 29–168 U/L	7,206	> 20,000	> 20,000	> 20,000	19,620	2,815	797	124	77
ALT 7–35 U/L	112	228	374	513	498	271	157	77	33
AST 9–45 U/L	–	3,175	1,776	1,440	510	87	40	33	28

AST = aspartate transferase; ALT = alanine transaminase; U/L = unit per liter. Normal ranges are indicated for a 2-year-old child.

Her hyperkalemia improved with polystyrene sulfonate and salbutamol, and kidney function began to improve on day four without dialysis. Normal values of serum creatinine and CK were reached on days 9 and 12, respectively.

Hematologically, thrombocytopenia and dilution anemia occurred with massive hydration and improved with decreasing infusion rate and resumption of diuresis.

Finally, our patient left the hospital without sequelae after 18 days of clinical surveillance and biological monitoring, including the first 6 days in the ICU.

## DISCUSSION

In French Guiana, located within the Intertropical Convergence Zone, all local bees have been Africanized since the arrival of the “killer bees” in 1975.^1^

The hybridization phenomenon did not change the composition of their venom,^12^ which is a complex cocktail of 102 proteins and peptides.^13^ To date, no antivenom has been marketed despite active research at the adult clinical trial stage.^14^

The major problem with “killer bees” is their swarm attacks involving hundreds of individuals. This specific behavior is explained by the release of a pheromone (2-heptanone) onto the skin of their target, in addition to that injected via the venom, thus emitting a powerful alarm signal.^15^

Children, because of their young age and low weight, are at high risk of dangerous consequences from an attack of a bee swarm. Their ability to flee, protect, or alert is all the more reduced the lower their age. In addition, the lower their weight, the more serious the envenomation will be.

Thus, in 2018, Schmidt defined a scale of morbidity and mortality according to the weight of the victim and the number of stings, as well as a median lethal dose of 19 stings/kg.^16^ Our patient received 10 stings/kg. According to Schmidt, this is a “very high” level of envenomation.

In children as in adults, such envenomation can lead to a biphasic systemic reaction with, at first, an immediate anaphylactic-type reaction (involvement of the skin-mucosal tissue, respiratory compromise, reduced BP or associated symptoms, and persistent gastrointestinal symptoms),^17,18^ followed by a toxic reaction (rhabdomyolysis), which can prove fatal because of acute renal failure (ARF) within 72 hours (Table 2).^12,19–24^ The pathophysiological mechanisms of this ARF include, on the one hand, prerenal hypovolemia resulting from a decrease in vascular resistance due to anaphylaxis, as well as tubular lesions due to rhabdomyolysis and the potential direct nephrotoxicity of melittin (approximately 50% of the dry weight of bee venom).^19,25,26^

**Table 2:** Key points of massive envenomation by killer bees (more than 1 sting/kg)

	Systemic reaction	
	Immediate	Delayed
Diagnosis	• Anaphylaxis:	• Rhabdomyolysis
	⇒ Involvement of the skin-mucosal tissue,	• Acute renal failure
	⇒ Respiratory compromise,	
	⇒ Reduced BP or associated symptoms,	
	⇒ Persistent gastrointestinal symptoms.	
Treatment recommended by learned societies	• Epinephrine IM, 10 µg/kg, repeated every 10–15 minutes until there is a response,	• Isotonic saline hydration.
	• Vascular filling,	
	• Oxygen therapy if needed.	

In our case, the immediate combination of clinical signs (face edema, diarrhea, and vomiting) makes it possible to diagnose anaphylaxis, according to the criteria put forward by Sampson^17^ and based on American and European guidelines.^27–29^

This severe reaction with no prior exposure was caused by, on the one hand, the direct action of the biogenic amines in the venom, and, on the other hand, the mast cell degranulation caused by melittin and the “mast cell degranulation peptide” in the venom.^12,19^

In line with American and European guidelines, early management by intramuscular injection of epinephrine, at a dose of 10 µg/kg for a child (repeated every 10–15 minutes until there is a response),^30^ can block the activation of mast cells, reduce the cascade of mediators of anaphylaxis, and increase vascular resistance.

However, epinephrine is regularly underused; according to Worm, only around 20% of European anaphylaxis cases receive epinephrine,^31^ unlike corticosteroids and antihistamines, which do not constitute emergency treatment.^32^

Our patient did not receive epinephrine. She became anuric at hour 18, and her first blood test shows ARF occurring before the peak of CK.

As recommended, the anaphylactic reaction should be monitored for at least 6 hours after the symptoms disappear.^27–29,31^ In the case of an envenomation of more than one bee sting/kg,^9^ this monitoring period will also make it possible to detect the second phase of the reaction.

Indeed, melittin and phospholipase A2 work by altering the integrity of cell membranes,^19^ usually causing severe rhabdomyolysis, complicated by ARF.^4,6,10,11,19–26^ According to Szugye,^33^ the intracellular contents of dead muscle cells being released into the extracellular space and circulating are responsible for kidney damage due to the following pathophysiological mechanisms: first, the activation of the renin–angiotensin–aldosterone system, which reduces renal blood flow; second, myoglobin (an oxygen-binding heme protein) is released in overwhelmingly high amounts and precipitates in the glomerular filtrate, causing damage to the renal tubules; and third, uric acid is released and forms crystal deposits causing tubular destruction.

In our case, the blood CK level remained very high during the first 4 days (> 20,000 U/L). Guidelines for the management of pediatric rhabdomyolysis do not currently exist, but, according to the Szugye review,^33^ isotonic saline is the most commonly used for hydration.

Our patient thus experienced ARF initially because of poorly treated anaphylaxis, which was later aggravated by rhabdomyolysis. She suffered a very common treatment error by not receiving epinephrine immediately. Immediate injection of epinephrine, while maintaining renal function during the initial anaphylactic phase, would have prevented blood accumulation of myoglobin and uric acid due to rhabdomyolysis. The renal tubules could thus have been preserved.

## CONCLUSION

In French Guiana, remote villages are only accessible by plane or canoe. In this context, isolated physicians should be aware of the signs of anaphylaxis. In such situations, they should administer epinephrine along with vascular filling as soon as possible to quickly halt a potentially fatal toxic chain reaction.

Then, during the first 24 hours, clinical surveillance and biological monitoring in an ICU is essential for the early detection of hypovolemic shock and respiratory distress.

Last, even if the initial reaction appears to be clinically resolved, the direct toxicity of the venom at a dose greater than 1 sting/kg can lead to ARF due to rhabdomyolysis. This justifies attentive medical surveillance for at least 72 hours.

## References

[1] Winston ML, 1992. The biology and management of Africanized honeybees. Annu Rev Entomol 37: 173–193.

[2] Keer WE, 1967. The history of the introduction of African bees in Brazil. Afr Bee J 39: 3–5.

[3] Michener CD, 1975. The Brazilian Bee problem. Annu Rev Entomol 20: 399–416. 1090242 10.1146/annurev.en.20.010175.002151

[4] Pucca MB Cerni FA Oliveira IS Jenkins TP Argemí L Sørensen CV Ahmadi S Barbosa JE Laustsen AH, 2019. Bee updated: current knowledge on bee venom and bee envenoming therapy. Front Immunol 10: 2090. 31552038 10.3389/fimmu.2019.02090 PMC6743376

[5] Costa AG Chaves BA Murta FLG Sachett JAG Sampaio VS Silva VC Monteiro WM, 2018. Hymenoptera stings in Brazil: a neglected health threat in Amazonas state. Revista da Socieda de Brasileira de Medicina Trop 51: 80–84. 10.1590/0037-8682-0109-2017 29513849

[6] Rahimian R Shirazi FM Schmidt JO Klotz SA, 2020. Honeybee stings in the era of killer bees: anaphylaxis and toxic envenomation. Am J Med 133: 621–626. 31715166 10.1016/j.amjmed.2019.10.028

[7] Marques MRV Araújo KAM Tavares AV Vieira AA Leite RS, 2020. Epidemiology of envenomation by Africanized honeybees in the state of Rio Grande do Norte, northeastern Brazil. Rev Bras Epidemiol 23: e200005. 10.1590/1980-549720200005 32130394

[8] Diniz AG Belmino JF Araújo KA Vieira AT Leite Rde S, 2016. Epidemiology of honeybee sting cases in the state of Caera, northeasterne Brazil. Rev Inst Med Trop Sao Paulo 58: 40. 27253742 10.1590/S1678-9946201658040 PMC4879997

[9] Bousquet J Coulomb Y Robinet-levy M Micel FB, 1982. Clinical and immunological surveys in beekeapers. Clin Allergy 12: 331–342. 7116611 10.1111/j.1365-2222.1982.tb02537.x

[10] Hommel D Bollondard F, 1998. Multiple African honeybee stings and acute renal failure. Nephron 78: 235–236. 9496750 10.1159/000044923

[11] Le Borgne A, 1998. Envenimations par piqûres multiples d’abeilles africanisées: revue à propos de cinq cas hospitalisés dans le service de réanimation du C. H. de Cayenne. Thèse de doctorat en médecine, Faculté de Brest, France.

[12] Schumacher MJ Egen NB, 1995. Significance of Africanized bees for public health. A review. Arch Intern Med 155: 2038–2043. 7575061

[13] Van Vaerenbergh M Debyser G Devreese B de Graaf DC, 2014. Exploring the hidden honeybee (*Apis mellifera*) venom proteome by integrating a combinatorial peptide ligand library approach with FTMS. J Proteomics 99: 169–178. 24606962 10.1016/j.jprot.2013.04.039

[14] Barbosa AN 2017. A clinical trial protocol to treat massive Africanized honeybee (*Apis mellifera*) attack with a new apilic antivenom. J Venom Anim Toxins Incl Trop Dis. 23: 14. 28331487 10.1186/s40409-017-0106-y PMC5356296

[15] Faita MR Colman Carvalho RMM Alves-Junior VV Chaud-Netto J, 2014. Defensive behavior of africanized honeybees (hymenoptera: apidae) in Dourados-Mato Grosso do Sul, Brazil. Revista Colombiana de Entomología 40: 235–240.

[16] Schmidt JO, 2018. Clinical consequences of toxic envenomation by Hymenoptera. Toxicon 150: 96–104. 29782951 10.1016/j.toxicon.2018.05.013

[17] Sampson HA 2006. Second symposium on the definition and management of anaphylaxis: summary report—second national institute of allergy and infectious disease/food allergy and anaphylaxis network symposium. J Allergy Clin Immunol 117: 391–397. 16461139 10.1016/j.jaci.2005.12.1303

[18] Ring J Behrendt H, 1999. Anaphylaxis and anaphylactoid reactions. Clin Rev Allergy Immunol 17: 13. 10.1007/BF02737644 10829809

[19] Hughes RL, 2019. A fatal case of acute renal failure from envenoming syndrome after massive bee attack: a case report and literature review. Am J Forensic Med Pathol 40: 52–57. 30531211 10.1097/PAF.0000000000000451

[20] West PL McKeown NJ Hendrickson RG, 2011. Massive hymenoptera envenomation in a 3-year-old: Pediatr Emerg Care 27: 46–48. 21206257 10.1097/PEC.0b013e3182045f47

[21] Tumwine JK Nkrumah FK, 1990. Acute renal failure and dermal necrosis due to bee stings: report of a case in a child. Cent Afr J Med 36: 202–204. 2282653

[22] Ariue B, 1994. Multiple Africanized bee stings in a child. Pediatrics 94: 115–117. 8008518

[23] Betten DP Richardson WH Tong TC Clarck RF, 2006. Massive honeybee envenomation-induced rhabdomyolysis in an adolescent. Pediatrics 117: 231–235. 16396886 10.1542/peds.2005-1075

[24] Bresolin NL Carvalho FC Goes JC Fernandes V Barotto AM, 2002. Acute renal failure following massive attack by Africanized bee stings. Pediatr Nephrol 17: 625–627. 12185470 10.1007/s00467-002-0888-0

[25] Grisotto LS Mendes GE Castro I, 2006. Mechanisms of bee venom-induced acute renal failure. Toxicon 48: 44–54. 16774771 10.1016/j.toxicon.2006.04.016

[26] Malik GH, 1998. Rhabdomyolysis and myoglobin-induced acute renal failure. Saudi J Kidney Dis Transplant 9: 273–284. 18408300

[27] Campbell RL Hagan JB Manivannan V Decker WW Kanthala AR Bellolio MF Smith VD Li JTC, 2012. Evaluation of national institute of allergy and infectious diseases/food allergy and anaphylaxis network criteria for the diagnosis of anaphylaxis in emergency department patients. J Allergy Clin Immunol 129: 748–752. 22051698 10.1016/j.jaci.2011.09.030

[28] Muraro A Roberts G Worm M Bilò MB, 2014. Anaphylaxis: guidelines from the European academy of allergy and clinical immunology. Allergy 69: 1026–1045. 24909803 10.1111/all.12437

[29] Gloaguen A Cesareo E Vaux J Valdenaire G Ganansia O Renolleau S Pouessel G Beaudouin E Lefort H Meininger C, 2016. Prise en charge de l’anaphylaxie en médecine d’urgence. Recommandations de la Société française de médecine d’urgence (SFMU) en partenariat avec la Société française d’allergologie (SFA) et le Groupe francophone de réanimation et d’urgences pédiatriques (GFRUP), et le soutien de la Société pédiatrique de pneumologie et d’allergologie (SP2A). Ann françaises de médecine d’urgence 6: 342–364.

[30] Ring J Klimek L Worm M, 2018. Adrenaline in the acute treatment of anaphylaxis. Dtsch Arztebl Int 115: 528–534. 30149833 10.3238/arztebl.2018.0528 PMC6131363

[31] Worm M 2014. First European data from the network of severe allergic reactions (NORA). Allergy 69: 1397–1404. 24989080 10.1111/all.12475

[32] Simons FE 2014. International consensus on (ICON) anaphylaxis. World Allergy Organ J 30: 9. 10.1186/1939-4551-7-9 PMC4038846 24920969

[33] Szugye HS, 2020. Pediatric rhabdomyolysis. Pediatr Rev 41: 265–275. 32482689 10.1542/pir.2018-0300

